# Precision post‐translational modification of PML defines neuroblastoma clinical behaviour

**DOI:** 10.1002/ctm2.70763

**Published:** 2026-08-18

**Authors:** Sreenidhi Mohanvelu, Poorvi Subramanian, Sheeja Aravindan, Madhi Oli Ramamurthy, Sivaroopan Aravindan, Afsana Parveen Jahir Hussain, Natarajan Aravindan

**Affiliations:** ^1^ Department of Physiological Sciences, College of Veterinary Medicine Oklahoma State University Stillwater Oklahoma USA; ^2^ OU‐Health Stephenson Cancer Center Oklahoma City Oklahoma USA

**Keywords:** high‐risk neuroblastoma, phosphorylation, PML, post‐translational modifications, pPML, promyelocytic leukaemia protein, spatiotemporal expression

## Abstract

**Background:**

Promyelocytic leukaemia protein (PML), a key regulator of nuclear architecture and cellular homeostasis, is increasingly recognised for its tumour‐suppressive functions. In neuroblastoma (NBL), a clinically heterogeneous and aggressive paediatric malignancy, the prognostic role of PML and its post‐translational modifications remains largely unexplored.

**Methods:**

We investigated the prognostic and predictive significance of PML expression and its site‐specific phosphorylation at serine 518 (S^518^) in a cohort of 121 NBL patients. Custom‐synthesised antibodies and high‐resolution tissue microarrays were used to quantitatively profile PML and pPML S^518^ expression via automated immunohistochemistry and digital image analysis. Survival outcomes were assessed using Kaplan‐Meier and Cox regression analyses. Mechanistic insights were obtained using reverse‑engineered phospho‑mutant models enabling selective modulation of S518 phosphorylation.

**Results:**

Our findings reveal a striking inverse relationship between PML abundance and S^518^ phosphorylation, with low PML and high pPML S^518^ strongly associated with advanced disease stage, metastasis, relapse, and therapy resistance. Survival analyses demonstrate that low PML predicts poor overall survival (OS), progression‐free survival (PFS), and relapse‐free survival (RFS), while elevated pPML S^518^ correlates with significantly worse outcomes across all endpoints. Multivariate Cox regression confirms both markers as independent predictors of survival. Mechanistically, reverse‐engineered phospho‐mutant models that enable selective switch‐on/switch‐off modulation of S^518^ phosphorylation demonstrated that phosphorylation at this site reduces PML abundance and promotes invasive cellular phenotype.

**Conclusion:**

PML loss and site‐specific phosphorylation of PML at S^518^ represent robust prognostic and predictive biomarkers with potential utility in risk stratification. Targeting PML phosphorylation may offer a promising translational strategy to improve therapeutic efficacy in high‑risk NBL.

**Key points:**

- PML depletion is a defining feature of aggressive neuroblastoma and is associated with advanced stage, metastasis and relapse.
- S^518^ phosphorylation emerges as the dominant post translational trigger committing PML to ubiquitin mediated degradation.
- High pPML S^518^ and low PML form a powerful prognostic axis that independently predicts OS, PFS, and RFS in neuroblastoma.
- PML loss and S^518^ phosphorylation define a molecular framework of heightened cellular plasticity underlying aggressive clinical behavior.

## Graphical abstract

- Promyelocytic leukaemia protein (PML) loss is significantly associated with advanced disease stage, metastasis, relapse, therapy resistance, and poor survival in neuroblastoma.
- Elevated phosphorylation of PML at S^518^ promotes PML destabilisation and predicts adverse clinical outcomes.
- The phosphorylated PML (pPML)/PML ratio provides enhanced prognostic stratification and more accurately reflects PML phosphorylation status.
- Phospho‐mutant studies demonstrate that S^518^ phosphorylation drives PML degradation and enhances invasive tumour behaviour.

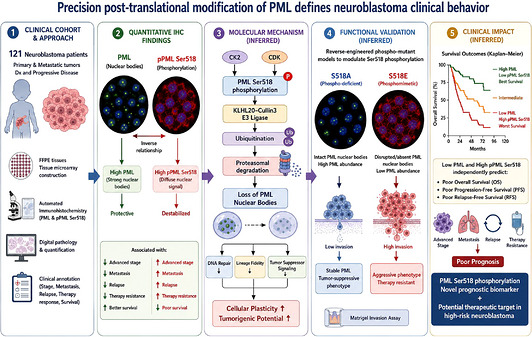

## 1 INTRODUCTION

Promyelocytic leukaemia protein (PML) is a nuclear hub interspersed throughout chromatin as heterogeneous nuclear structures called PML nuclear bodies (PML‐NBs).^8^, ^34^ They are the ‘command centres’ with adaptive regulatory modules orchestrating various protein‐protein interactions and facilitating diverse cellular and molecular processes such as cell cycle arrest, apoptosis, senescence, transcriptional regulation, DNA repair and intermediary metabolism.^6^, ^8^ By coordinating these complex cellular networks, PML‐NBs function as molecular switches that integrate intracellular cues to determine the balance between cellular self‐renewal and lineage commitment, thereby regulating cellular plasticity.^16^, ^19^, ^39^ This remarkable functional versatility, which is central to regulating trans‐differentiation, dedifferentiation and lineage priming, is orchestrated by two mutually reliant and interdependent components. (i) PML‐NBs structural integrity is often stabilised by intrinsic self‐assembly mediated by nucleation, dimerisation and polymerisation of PML through its RING finger, B‐box, coiled‐coil (RBCC)/tripartite motif (TRIM) to form PML‐NBs and further reinforced by multivalent interactions of small ubiquitin like modifier (SUMO)–SUMO interacting motif (SIM) motifs.^7^, ^33^ These multivalent networks not only stabilise the architectural core of PML‐NBs but also enable selective and reversible recruitment of client proteins, mediating precise spatial and temporal regulation of protein activity. (ii) PML‐NBs abundance is governed by regulatory dynamics of transcriptional machinery such as the signalling cascades of p53,^7^ interferon‐stimulated response elements (ISREs), retinoic acid (RA) receptors and oxidative response elements and several post‐translational modifications (PTMs) including SUMOylation, phosphorylation, acetylation and ubiquitination in response to external stress.^6^ Consequently, any disruption of the regulatory networks undermines the coordination of these key molecular mechanisms, tipping the fine balance between self‐renewal, pluripotency and quiescence prompting phenotypic plasticity that fosters tumour heterogeneity, therapeutic resistance and disease relapse. While the oncogenic fusion‐driven framework of PML‐NBs as PML‐RARα that typically impairs the assembly and integrity of PML in acute promyelocytic leukaemia (APL) patients is well characterised,^6^ its existence and function in solid tumours, in particular in deadly extracranial tumours in infants, neuroblastoma (NBL) is rare. Although, PML has been majorly regarded as the tumour suppressor owing to its established roles in maintaining genomic stability, promoting apoptosis, enforcing cellular senescence, and restricting malignant transformation, evidence also indicated that the biological functions of PML are highly context dependent. For instance, in some haematologic and solid malignancies, PML has been reported to support tumour maintenance, metabolic adaptation, stemness, and therapy resistance.^1^, ^3^, ^10^, ^14^ Studies in chronic myeloid leukaemia, glioblastoma, triple‐negative breast cancer, and other tumour types have demonstrated that PML can promote cancer cell survival and disease progression under specific molecular and microenvironmental conditions.^15^, ^20^, ^21^, ^27^, ^38^ These observations have led to the recognition that PML cannot be universally classified as either tumour suppressive or oncogenic; rather, its functional consequences appear to be dictated by cellular lineage, genetic background, post‐translational regulation, and disease context. Consequently, defining the tumour‐specific role of PML and the mechanisms governing its activity in NBL remains an important area of investigation.

Mechanistically, functional loss of PML‐NBs in solid tumours is primarily driven by ubiquitination and subsequent proteasome‐mediated degradation which disrupts the spatial organisation of PML‐NBs, impairing the cell's ability to maintain lineage fidelity and genomic integrity fostering a permissive environment for increased cellular plasticity.^33^ Phosphorylation serves as a key priming event in this degradation pathway in diverse cancers including lung, breast, prostate cancer and others.^13^, ^30^, ^36^ It alters the structural conformation of PML‐NBs, exposes the interaction motifs and creates docking sites for E3 ubiquitin ligase facilitating polyubiquitination, effectively committing PML‐NBs to proteasomal degradation. While phosphorylation of PML at serine 403 (S^403^) by ATR/ERK2; S^505^ and S^530^ by ERK; S^527^ and S^565^ by CK2 displayed minor involvement in PML degradation, strikingly, phosphorylation of evolutionarily conserved S^518^ by CK2 or CDK1/2 serves as the major regulatory site for PML degradation through the initiation of ubiquitination cascade.^4^, ^23^, ^36^ Unlike the other phosphorylation sites, S^518^ independently prompts KLH20‐Cullin3‐mediated degradation of PML.^36^ However, despite these insights, the cross‐talk with other PTMs, downstream functional consequences and most importantly its clinical relevance remain largely elusive in solid tumours and yet to be delineated in NBL, a clinically heterogenous paediatric malignancy. Based on the established function of S^518^ phosphorylation in promoting PML degradation, we hypothesised that pPML‐S^518^ may represent a critical regulatory mechanism underlying the reduced PML expression associated with aggressive NBL.

NBL is an aggressive cancer in infants that originates from the neural crest cells during development, globally accounting for 8%–10% of infant cancers and contributing to 15% of paediatric cancer mortalities.^26^ The clinical manifestation of the disease spans a broad spectrum, ranging from most severe and persistent forms to well‐controlled cases with favourable outcomes. Patients with high‐risk disease typically undergo intensive multi‐modal clinical therapy (IMCT). The therapeutic regimen is structured into sequential phases of induction therapy with tumour resection surgery and chemotherapy (cisplatin, etoposide, vincristine, cyclophosphamide, doxorubicin, and topotecan) followed by a consolidation phase of high‐dose chemotherapy, radiotherapy and haematopoietic stem cell rescue.^24^ This is subsequently followed by maintenance phase, which aims to sustain remission and prevent relapse, often incorporating retinoid drug (isotretinoin), immune activating cytokines (GM‐CSF and IL‐2) and monoclonal antibody therapy (dinutuximab, naxitamab). Despite these refined therapeutic strategies, >50% of the patients experience recurrences, an additional 10%–20% of the patients’ relapse with tumours that are refractory to further treatment. These clinical outcomes not only highlight the therapeutic limitations but points to a deeper biological complexity driving therapy resistance, disease persistence and progression.

One of the most enduring treatments in the therapeutic framework is RA treatment that still remains a cornerstone, due to its ability to induce differentiation in the residual tumour. However, its therapeutic efficacy largely hinges on the stability/abundance of PML‐NBs for orchestrating RA‐driven signal transduction. Recognising the pivotal role of PML‐NB in therapy response and the centrality of S^518^ phosphorylation, herein, we investigated the predictive and prognostic significance of constitutive and site (S^518^)‐specific phosphorylation of PML in a cohort of (*n* = 121) NBL patients. The outcomes displayed a strong inverse relationship between PML availability and its S^518^ site‐specific phosphorylation. Crucially, this study identified a low‐is‐worse association for PML; and a high‐is‐worse association for PML phosphorylation at S^518^ to the disease progression and clinical outcomes for children presented with NBL. Further the outcomes suggest that phosphorylation of PML at S^518^ is not merely a priming event for PML destabilisation, rather a regulator of enhanced cellular plasticity, contributing to NBL progression, relapse and unfavourable clinical outcomes.

## 2 MATERIALS AND METHODS

### 2.1 Cell culture

All cell lines used in the study are well‐characterised, and de‐identified human‐derived models and all experimental procedures were performed in accordance with institutional guidelines and regulatory standards governing the use of de‐identified, commercially sourced human cell lines. A panel of HR‐NBL, stage 4 human cell lines were obtained from the Children's Oncology Group (COG) NBL cell line repository (Lubbock) and were cultured as described in our previous studies.^31^ Diagnostic‐stage (Dx) cell lines included primary tumour‐derived CHLA‐15 and metastatic CHLA‐42 and SH‐SY5Y cells. Progressive disease (PD) cell lines included primary tumour‐derived CHLA‐20 and NB‐EB‐C1, as well as aggressive metastatic CHLA‐90, CHLA‐60, CHLA‐61, CHLA‐79, CHLA‐140, CHLA‐171, CHLA‐172, LA‐N‐6, and SK‐N‐FI cells. All cell lines were authenticated by COG and are available online (http://www.cogcell.org/clid.php).^32^ In brief, cells were cultured in IMDM supplemented with 20% FBS, 4 mM L‐glutamine, 5 µg/mL insulin, 5 µg/mL transferrin, 5 ng/mL selenous acid and 1% Pen‐strep (penicillin, 12 units/mL; streptomycin, 12 µg/mL). LA‐N‐6 and SK‐N‐FI cells were cultured and maintained in RPMI‐1640 medium supplemented with 10% FBS and 1% Pen‐strep. For passage and all other experiments, the cells were detached using trypsin (.05%) and EDTA (.53 mM; Corning), re‐suspended in complete media and incubated in a 95% air/5% CO_2_ humidified chamber. Cell viability was performed using .4% trypan blue exclusion (Sigma) and cells were counted using the countess automated cell counter (Invitrogen).

### 2.2 Phospho‐engineering PML Ser^518^ NBL clones

Phospho‐deficient (PML‐S518A) and phosphomimetic (PML‐S518E) NB models were generated to examine the functional consequences of PML Ser^518^ phosphorylation. PML‐S518A mutants were established in the PD clones CHLA‐20 and CHLA‐90, whereas PML‐S518E mutants were generated in Dx clones CHLA‐42 and CHLA‐15. Cell lines were strategically selected to capture distinct biological and clinical contexts relevant to phosphorylation of PML at Ser^518^, including differences in PML expression, tumour origin, disease stage, and treatment‐associated progression. Both PML S518A and PML S518E expression constructs were custom synthesised and cloned into the pcDNA3.1(+)‐C‐DYK backbone (GenScript). The integrity of the engineered codon substitutions was confirmed by restriction enzyme digestion and sequencing (GenScript). Patient‐derived clones were transfected using Neon Nxt electroporation transfection system with pulse voltage (1200 V), width (20 ms) and two pulses (Life Technologies) and stable clones were selected using G418 (200 µg/mL; Selleckchem). Modifications of phosphorylation status in generated clones were validated by immunoblotting and compared to their respective parental controls.

### 2.3 Immunoblotting

Cellular protein extraction, quantitation, and immunoblotting were performed as described in our earlier studies.^25^ In this study, to characterise PML expression, phosphorylation at S^518^, and SUMOylation states across Dx and PD NBL models, blots were incubated with our custom‐synthesised PML, S^518^ site‐specific phosphorylated PML antibodies (Human reactivity; Abclonal) and PML SUMOylation site‐specific antibody (Thermo Scientific). All membranes were developed using appropriate anti‐rabbit secondary antibodies (Invitrogen). Quantitation of blots was performed using BioRad Quantity One gel analysis software. GAPDH, α‐tubulin, or β‐actin normalised values were compared between groups and statistical tests involving *t*‐tests and/or ANOVA with post hoc correction were performed in GraphPad Prism. A *p* ≤ .05 was considered statistically significant.

### 2.4 Tumour cell invasion

Cell invasion assays were performed as described in our earlier studies.^31^ Functional alterations in invasive potential of PML S518A expressing CHLA‐20, CHLA‐90 and PML S518E expressing CHLA‐42 (vs. their parental controls) were assessed using Boyden invasion assay under proliferation‐controlled (mitomycin C, 10 µg/mL, Sigma) conditions. Polycarbonate inserts containing 8 µm pores were pre‐coated with 50% Matrigel as described previously. Cells were seeded into the upper chamber in serum‐free medium, while the lower chamber contained complete medium with chemoattractant. After 24 h of incubation, non‐invading cells were removed from the upper membrane surface. Cells that invaded through the Matrigel and reached the lower surface were fixed in methanol: acetic acid (3:1) and stained with .1% crystal violet. Images were acquired at .8× magnification and quantified using ImageJ across multiple fields to ensure representative sampling. Group‐wise comparisons were performed using GraphPad Prism and presented as mean ± SEM. Statistical significance was determined using one‐way ANOVA, with thresholds defined as *p < .05, **p < .01, ***p < .001, ****p < .0001, and ns (not significant).

### 2.5 NBL specimen collection and patient demographics

A total of 140 clinical biospecimens from 121 cases of human NBL were collected from University of Oklahoma Health Sciences Center (OUHSC) paediatrics pathology collection. Additional specimens (nine from nine cases) were procured from NCI‐Cooperative Human Tissue Network (CHTN) and Oregon Health Science University Biospecimen core. A specimen and/or case‐matched demographics are obtained (Table 1). All protocols were approved by the OUHSC and Oklahoma State University (OSU) Institutional Review Boards (IRB), with permission for the research use of de‐identified specimens. All experiments were performed in accordance with our institutions’ IRB guidelines and regulations for the protection of human subjects. The cohort comprised patients with a definitive Dx of NBL and with adequate pathological and clinical data for analysis. Inclusion criteria included well‐documented demographic and clinical variables including stage (INSS I–IV) at Dx, age, tumour site (primary or metastatic), relapse and/or recurrence history, disease status (treatment naïve disease at Dx or IMCT defying PD), risk status, and more importantly alive/dead status matched with survival in months. Specimens with insufficient clinical or pathological information or survival outcomes were excluded from the study. These criteria ensured a well‐defined cohort suitable for retrospective evaluation of prognostic factors and survival outcomes. A cohort of 121 NBL patients satisfied the inclusion/exclusion criteria were included for PML expression and phosphorylation (S^518^) analysis and subsequent correlation studies.

**TABLE 1:** Demographics of neuroblastoma patients.

Total cases	*n* = 121 (Survival data available *n* = 95)			
Total specimens	*n* **=** 140			
Gender	Male *n* **=** 70		Female *n* **=** 51	
Age	≤2Y *n* = 53		>2Y *n* = 68	
N‐Myc status	Amplified *n* = 17		Non‐amplified *n* = 104	
INSS stage	I *n =* 19	II *n* = 13	III *n* = 18	IV *n* = 46
Tumour site	Primary *n =* 40	Metastatic *n =* 35		
Relapse	No relapse *n =* 50	Relapse *n =* 32		
Survival	Live *n =* 59	Dead *n =* 36		
Disease status	Diagnosis *n =* 45	PD‐IMCT *n =* 43		

### 2.6 Tissue microarray construction

For this, first the haematoxylin and eosin (H&E) stained sections from the FFPE blocks of NBL specimens were screened by paediatric pathologist, and the specimens holding sufficient percent tumour with minimal necrosis, and adequate tumour volume were included for the tissue microarray (TMA) construction. TMA construction executed in the tissue pathology core of OU Health Stephenson Cancer Center (OU‐SCC) following the standard protocols as described in our previous study.^2^ In brief, the cores from 10% NBF‐fixed and paraffin‐embedded (FFPE) tissues were non‐sequentially positioned (in triplicate of 1 mm cores) on the recipient block. The triplicate sampling approach was employed to account for intra‐tumoural heterogeneity and enhance the reliability of staining and other downstream applications. Following array construction, the TMA blocks were baked and sectioned at 4 µm thickness and subsequently utilised for immunohistochemistry (IHC). Cases with missing cores or non‐representative cores were flagged and excluded from the analysis. Custom archival of specimens in the TMAs ensures unbiased staining and expression analysis across the cohort.

### 2.7 Custom synthesis of purified PML and S^518^ site‐specific phosphorylated PML antibodies

Custom PML and S^518^ site‐specific phosphorylated PML antibodies were synthesised in collaboration with ABclonal Technology using the synthetic peptide antigen corresponding to amino acids 514–522 of human PML. In brief, the phosphorylated peptide (SKAV[pS]PPHL) and its non‐phosphorylated counterpart (SKAVSPPHL) were synthesised at >85% purity (validated by HPLC and MS) and were conjugated to keyhole limpet hemocyanin (KLH) for enhanced immunogenicity. New Zealand white rabbits were immunised following a 5‐day schedule over a period of 54 days, beginning with a primary dose of .7 mg emulsified in Freud's adjuvant, followed by 5 booster doses of .35 mg in incomplete Freud's adjuvant. Post‐immunisation sera were collected and purified utilising antigen affinity chromatography. The resulting antibodies were quantified and dot blot assays confirmed strong reactivity of purified antibodies against phosphorylated peptide with minimal cross reactivity with non‐phosphorylated peptide. Further to validate the specificity of the custom‐generated antibodies against total PML and phosphorylated PML (pS^518^‐PML), peptide competition IHC was performed on representative human NBL tissue sections. Prior to tissue incubation, each antibody was pre‐incubated with increasing concentrations of its corresponding immunising peptide (PML peptide for total PML antibody and phospho‐S^518^ peptide for pS^518^‐PML antibody). Sections stained with antibodies alone exhibited strong nuclear immunoreactivity, whereas pre‐incubation with the cognate peptide resulted in a concentration‐dependent reduction and eventual loss of staining signal. The phospho‐specific antibody was similarly competed by the phosphorylated peptide, confirming selective recognition of the phosphorylated epitope and minimal nonspecific binding. These experiments demonstrated the specificity of both antibodies for their intended targets and provided validation for their use in subsequent IHC analyses. Representative results are shown in Figure S1.

### 2.8 PML and pPML^S518^ immunohistochemistry

For IHC purposes, custom‐designed purified PML and pPML antibodies were optimised for concentration, antibody‐specific labelling, and so forth. IHC for PML and pPML was performed utilising Leica Bond RX fully automated staining systems (Leica Biosystem Inc., Buffalo Grove, IL; Leica Bond III) according to the manufacturer's protocol using the Bond Polymer Refine detection system at the tissue pathology core of OU‐SCC. In brief, TMA sections (4 µm thickness) were sequentially baked, rehydrated, neutralised with .25 M Tris–HCl, followed by blocking (.5% BSA–2% FBS). The sections were then labelled with our rabbit polyclonal anti‐human PML and pPML S^518^ antibodies. A peroxidase‐diaminobenzidine visualisation process was employed, which gave positive immunoreactivity a brown colour. Appropriate tissue histology controls stained with H&E stain and negative controls with isotype matched (rabbit IgG Isotype control, ThermoFisher Scientific) no primary antibody were examined in parallel. The slides were digitally scanned into virtual slides at high‐resolutions (40×) using Zeiss Axioscan.Z1, equipped with Calibre7, a high‐performance slide scanner that enables whole‐slide imaging with advanced control over acquisition parameters ensuring consistent resolution and colour fidelity. The integrated Zeiss‐Zen software enables post‐acquisition image optimisation, noise suppression, contrast and brightness adjustments and illumination corrections enhancing image quality and reliability for downstream analysis.

### 2.9 Quantitative profiling of PML expression and phosphorylation (pPML) at S^518^

Digitised TMA were analysed for nuclear localisation and positive expression of PML and its phosphorylation at S^518^ using HALO image analysis platform (Indica Labs), a pioneering tool in digital pathology that enables high throughput, quantitative assessment of the expression patterns. HALO's machine learning based algorithm facilitated accurate segmentation and object quantification, including the automatic detection of regions of interest. The Multiplex IHC module was employed to precisely evaluate the nuclear DAB staining and haematoxylin counterstain with key parameters such as nuclear detection, nuclear aggressiveness, nuclear and cytoplasmic radius and optical density thresholds. Nuclear segmentation was achieved based on the haematoxylin detection algorithm that were optimised to .53 to separate individual nuclei in the densely packed regions. To accurately eliminate microdebris and abnormally large, potentially fused nuclei, nuclear thresholds were accurately defined in the range 6 µm^2^ to 220.7 µm^2^. A circularity filter of .1–1.0 was applied to accurately capture reasonably irregular nuclei. Additionally, solidarity constraints were implemented to exclude segmentation defects and internal discontinuities that favour well‐defined, compact nuclei. The optical density thresholds were determined using control tissues and used across all the TMA's to empirically determine negative, weak, moderate, and strong positive nuclei for either PML or pPML S^518^. Quantitative outputs including the percentage of positive nuclei, mean optical density of nuclei and distribution profile of the staining intensities, were exported for statistical analysis. To ensure unambiguous interpretation and minimise variability, only cells exhibiting strong nuclear positivity were considered for analysis. This digital workflow ensures reproducibility and eliminates observer bias by standardising image evaluation across the cores.

### 2.10 Statistical analysis

For all group‐wise comparisons and clinical outcome (Overall survival, OS; Progression‐free survival, PFS; Relapse‐free survival, RFS) analysis, GraphPad Prism (version 10.1.0, GraphPad Software) was used, and for the Cox Proportional Hazards model (CPHM) statistical analysis (XL STAT Advanced 2025.1, Lumivero) was used. Survival analysis was performed using log‐rank (Mantel–Cox) test and assessed the chi‐square survival distributions and log‐rank hazard ratio by constructing Kaplan–Meier plots. We generated a CPHM statistical analysis, a well‐recognised statistical regression method for survival analysis that deals with time‐to‐event data and analyses the relationship between the survival outcome of a patient and several explanatory variables simultaneously. This model enables the quantification of the risk of an event occurring at any given time based on hazard ratio (HR) and the evaluation of changes in the levels of multiple independent factors that offer clinically meaningful insights into a patient's survival and prognosis. For the group‐wise comparisons, survival distributions, and for CPHM, a *p* value of <.05 is considered statistically significant.

## 3 RESULTS

### 3.1 PML loss predicts poor prognosis

**FIGURE 1:**
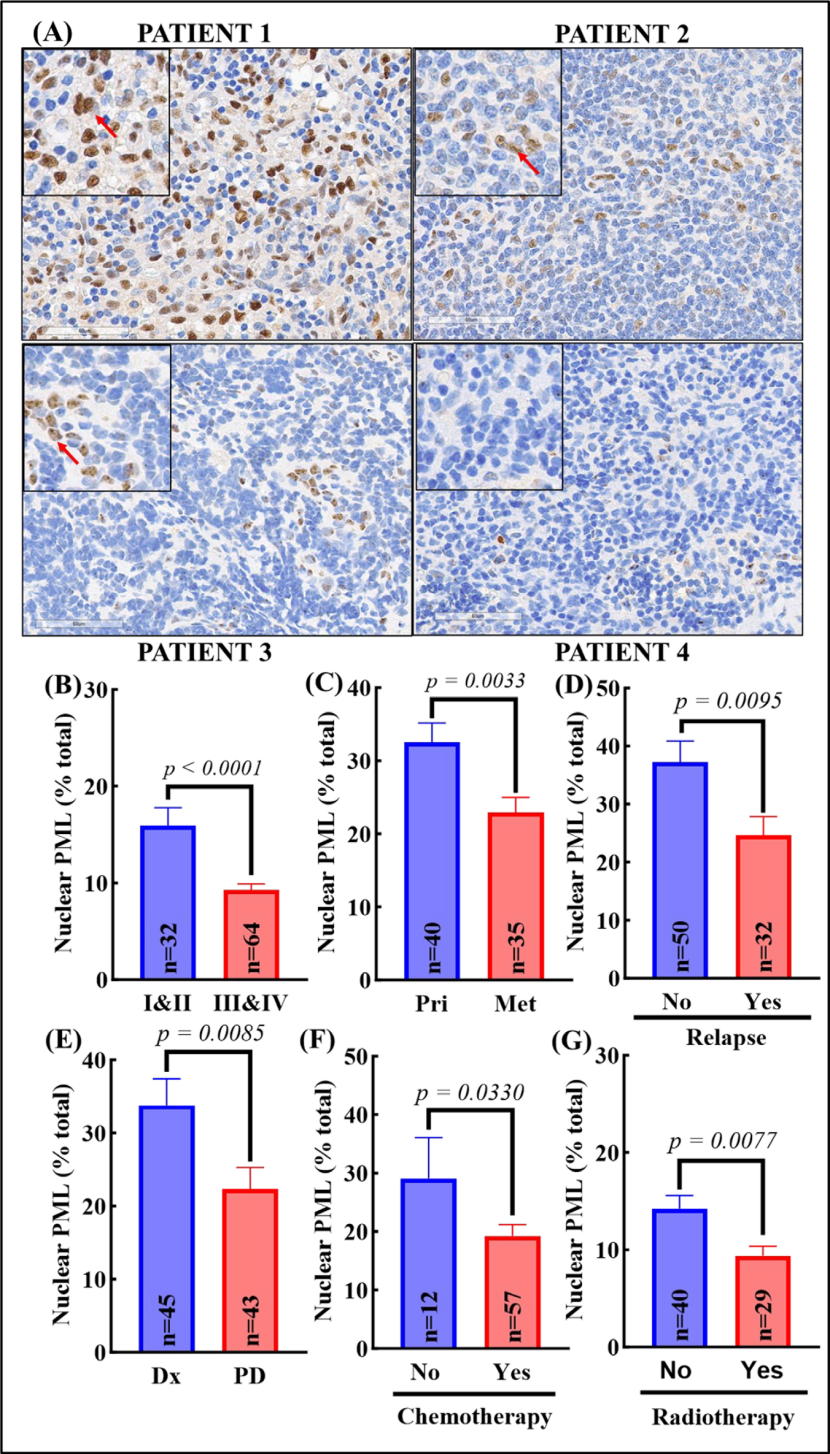
Promyelocytic leukaemia protein (PML) abundance predicts poor neuroblastoma (NBL) disease prognosis: (A) Representative microphotographs showing PML nuclear abundance in NBL patients (Patients 1–4). The red arrows in the insert images highlight the punctate nuclear expression of PML (Insert magnification: 20×). Histograms from the patient cohort (*n* = 121) showing loss of nuclear PML at (B) advanced disease stage compared with other INSS (*p* < .0001); (C) metastatic versus primary tumour site (*p* = .0033); (D) relapsed disease versus non‐relapsed (*p* = .0095); (E) intensive multi‐modal clinical therapy defying (PD) versus therapy naïve (Dx) tumours (*p* = .0085); (F) chemotherapy administered versus chemotherapy naïve cases (*p* = .0330); and (G) radiotherapy administered versus radiotherapy naïve cases (*p* = .0077).

PML expression in NBL patients was evaluated by IHC in a well‐annotated cohort of 121 patients. We analysed our custom archived TMAs comprising of a comprehensive panel of NBL tumours representing various clinical stages, anatomical sites, metastatic and disease (Dx/PD) status. To ensure representative sampling, three spatially independent cores were examined for each specimen. Immunohistochemical (IHC) analysis revealed distinct nuclear localisation of PML, characterised by predominantly discrete punctate structures and strong diffuse nuclear staining (Figure 1A). Although the characteristic distribution was consistent across the specimens, notably PML expression ranged from .142% to 99.594%, with a mean of 18.619% and median of 10.129%. Such a skewed distribution of PML indicates substantial loss of PML in majority of the tumours reflecting on the patterns of downregulation in NBL tumours. Consequently, we sought out to investigate the association, if any, of PML expression and the trajectory of NBL progression. Primarily, we examined the INSS stage‐dependent variations of PML expression. A pronounced and significant decline (*p* < .0001) in PML expression was evident in advanced stage tumours (stages III and IV) compared to early‐stage tumours (stages I and II; Figure 1B). The disease stage‐dependent PML deficiency recognises the function of PML in limiting tumour progression. Since, metastasis is a distinct phenotype that contributes to therapy resistance and relapse, and not merely a clinical stage, we assessed its significance. We observed a directly proportional loss of PML in metastatic tumours when compared with the primary tumours (*p* = .0033). Similarly, relapsed tumours displayed relatively low levels of PML (*p* = .0095) compared to favourable disease (Figure 1C,D). This loss of PML compromises the key regulatory mechanisms governing the cellular identity, thereby fostering a permissive state for phenotypic switching and enhanced plasticity that enables tumour cells to adopt invasive and metastatic traits facilitating their dissemination in hostile microenvironments. Prompted by this pattern, we investigated whether therapy‐driven PML loss potentially contributes to the emergence of treatment resistant phenotype. For this, comparing disease at Dx, we observed a significant (*p* = .0085) decrease of PML in PD (Figure 1E). Consistently, our analysis revealed a marked PML loss in patients who received chemotherapy (*p* = .0330) and radiotherapy (*p* = .0077) indicating either (i) selection or induction of adaptive resistance mechanisms under therapeutic pressure, or (ii) a failure of PML‐dependent tumour‐suppressive responses to therapy‐induced DNA damage due to PML destabilisation and functional disengagement (Figure 1F,G). Collectively, these observations underscore a strong association for PML loss to the NBL pathogenesis and prognosis, and suggest that PML loss is a definitive determinant that may actively contribute to tumour plasticity and therapeutic escape.

### 3.2 PML availability predicts clinical outcomes

**FIGURE 2:**
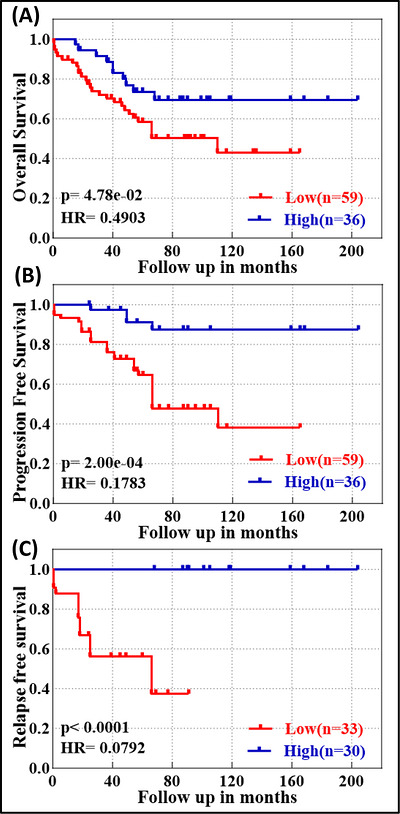
Promyelocytic leukaemia protein (PML) expression and neuroblastoma (NBL) clinical outcomes: Kaplan–Meier curves showing the association of the expression of PML with (A) overall survival (hazard ratio [HR]: .4903; 95% confidence interval [CI], .2557–.9399); (B) progression‐free survival (*n* = 95; HR: .1783; 95% CI, .08879–.3581); and (C) relapse‐free survival in a cohort of 63 NBL patients (HR: .0792; 95% CI, .02818–.2230). All survival analysis (log‐rank Mantel–Cox test and HR) and Kaplan–Meier curves were performed in GraphPad Prism. Blue = high PML; Red = low PML abundance.

To assess the influence of PML loss on the survival outcomes of NBL patients, we performed Kaplan–Meier survival analysis coupled with log‐rank (Mantel–Cox) test. Of the 121 patients, 95 patients with survival data were considered for analysis. Further, with the subset of relapse status availability, 63 patients were evaluated for RFS. The survival analysis was computed for cells expressing high and low (median, ≥14.29%) levels of nuclear PML. To ensure comprehensive representation and avoid potential bias, all tumour specimen available per patient, encompassing samples from multiple anatomical sites, as well as those collected from both Dx and PD stages were included. The cohort was predominated by patients with PML deficiency (*n* = 59), while 36 patients showed high PML. Survival outcomes were significantly stratified by PML expression across all the endpoints‐ OS, PFS and RFS. Low PML was consistently associated with adverse survival outcomes in contrast to high PML that showed better OS (*p* = 4.78e−02; HR: .4903), PFS (*p* = 2.0e−04; HR: .1783) and RFS (*p* < .0001; HR: .0792; Figure 2). An enhanced survival advantage was associated with PML expression, with median OS extending to 76 months as opposed to 57 months in low PML cases. Notably, this survival benefit extended beyond OS, with high PML associated with a pronounced delay in disease progression and longer remissions. To further determine whether therapy‐associated loss of PML expression contributes to disease progression and adverse patient outcomes, we evaluated survival endpoints within a subset of 53 NBL patients with documented prior treatment exposure. Despite experiencing comparable therapeutic interventions, patients exhibiting low PML expression demonstrated significantly worse clinical outcomes than those retaining higher PML levels. Kaplan–Meier survival analyses revealed that reduced PML expression was associated with significantly inferior OS (*p* = 4.50e−02; HR: 2.207; Figure S2A), indicating a substantial reduction in long‐term survival probability. Similarly, low PML expression correlated with markedly shortened PFS (*p* = 1.17e−02; HR: 8.458; Figure S2B), reflecting an increased likelihood of disease progression following treatment. RFS was likewise significantly compromised in the low‐PML group (*p* = 4.07e−02; HR: 6.488; Figure S2C), suggesting a heightened propensity for tumour recurrence. Collectively, these findings demonstrate that therapy‐associated depletion of PML is not merely a consequence of treatment‐induced tumour evolution but potentially represents a clinically significant molecular event linked to disease aggressiveness, recurrence, and diminished patient survival. More importantly, multivariable analysis using the CPHM confirmed PML as an independent predictor of survival in patients with NBL (*p* = .005, Wald *χ* ^2^ = 7.847; Table 2A). Multicollinearity was assessed using variance inflation factor (VIF) analysis, which demonstrated low VIF values for all covariates (Stage: 1.190; Age: 1.210; Gender: 1.080; MYCN status: 1.046), indicating minimal collinearity among predictors and supporting the robustness and stability of the Cox regression model (Table 2B). These findings further support the notion that reduced PML expression facilitates dynamic cellular reprogramming towards more aggressive and therapy‐resistant phenotypes, thereby enhancing tumour cell adaptability and contributing to poor clinical outcomes.

**TABLE 2:** Multivariate analysis: Cox proportional hazard model and the variance inflation factor (VIF) analysis of the Cox proportional hazard model in NBL patients.

(A)					
Variable	Value	Standard error	Wald chi‐square	Pr > chi‐square	Hazard ratio
**Stage**	1.057	.402	6.930	.008	2.879
**Age >=24m/<24m**	−2.044	.526	15.131	.000	.129
**Gender**	−.612	.396	2.385	.123	.542
**MYCN status**	.880	.383	5.276	.022	2.411
**PML**	−1.095	.391	7.847	.005	.335

(B)					
Multicollinearity statistics for PML					
	**Stage**	**Age**	**Gender**	**MYCN Status AM/NA**	
**VIF**	1.190	1.210	1.080	1.046	

(C)				
Variable	Value	Standard error	Wald chi‐square	Pr > chi‐square	Hazard ratio
Stage	.716	.344	4.334	.037	2.047
**Age >=24m/<24m**	−1.907	.518	13.553	.000	.149
**Gender**	−.692	.397	3.047	.081	.500
**MYCN status**	.963	.388	6.162	.013	2.620
**pPML S^518^**	.020	.010	4.256	.039	1.020

(D)					
Multicollinearity statistics for pPML S^518^					
	Stage	Age	Gender	MYCN status AM/NA	
**VIF**	1.215	1.196	1.054	1.057	

### 3.3 Single, site‐specific PTM, serine phosphorylation at 518 associates with disease prognosis

**FIGURE 3:**
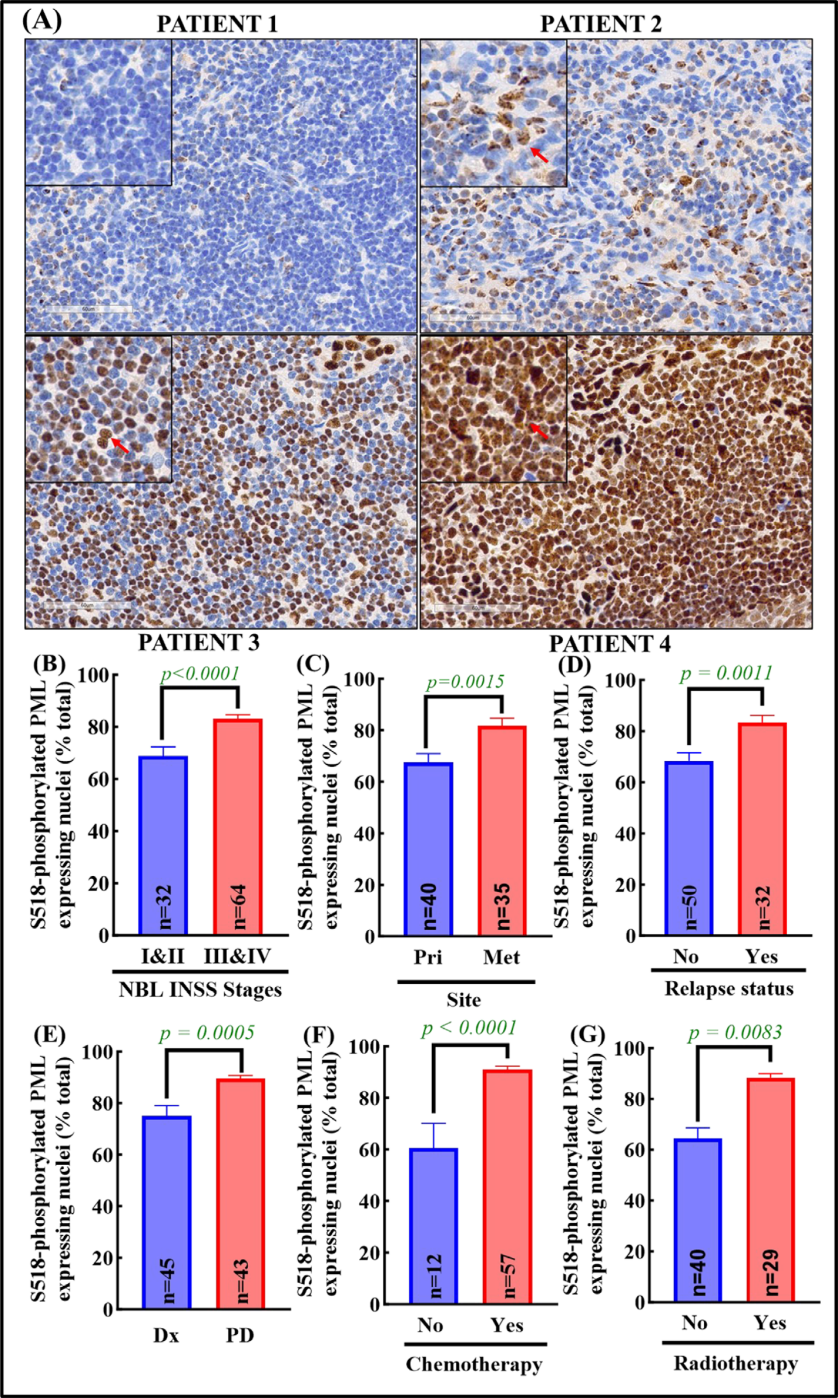
Association of phosphorylated promyelocytic leukaemia protein (pPML) S^518^ with neuroblastoma (NBL) disease prognosis: (A) Representative microphotographs showing pPML S^518^ nuclear abundance in NBL patients (Patients 1–4). The red arrows in the insert images highlight the diffused nuclear localisation of phosphorylated promyelocytic leukaemia protein (PML; Insert magnification: 20×). Histograms from the patient cohort (*n* = 121) showing high pPML S^518^; (B) advanced disease stage compared with other international neuroblastoma staging system (INSS; *p* < .0001); (C) metastatic versus primary tumour site (*p* = .0015); (D) relapsed disease versus non‐relapsed (*p* = .0011); (E) intensive multi‐modal clinical therapy defying (progressive disease [PD]) versus therapy naïve (Dx) tumours (*p* = .0005); (F) chemotherapy administered versus chemotherapy naïve cases (*p* < .0001); and (G) radiotherapy administered versus radiotherapy naïve cases (*p* = .0083).

While our findings establish reduced PML expression as a marker of poor clinical outcome in NBL, the molecular mechanisms underlying PML loss remain poorly defined. In multiple solid tumour types, functional depletion of PML‐NBs is driven primarily through post‐translational regulation involving phosphorylation‐dependent ubiquitination and proteasomal degradation. Among the several phosphorylation sites reported on PML, phosphorylation at the evolutionarily conserved serine 518 (S^518^) residue has emerged as the principal regulatory event that initiates KLHL20–Cullin3‐mediated ubiquitination and degradation of PML. Unlike other reported phosphorylation sites that exhibit relatively minor contributions to PML turnover, S^518^ phosphorylation can independently trigger the degradation cascade, leading to disruption of PML‐NB integrity and loss of PML‐mediated tumour‐suppressive functions. Given the established role of S^518^ phosphorylation as a critical determinant of PML stability, we next investigated the clinical distribution and prognostic significance of pPML‐S^518^ in NBL to determine whether this regulatory event contributes to the observed loss of PML and aggressive disease behaviour. To define the prognostic relevance of phosphorylation of PML at S^518^ (pPML S^518^), we systematically analysed the clinical relevance of this select PTM in our NBL patient's cohort (*n* = 121). Interestingly, pPML S^518^ in tumours ranged from 5.218% to 99.52% with a mean of 73.006% and a median of 78.879%, evidently recognising a substantial intertumoural heterogeneity. Localisation of pPML S^518^ displayed a diffused pattern in the tumours (Figure 3A). Owing to the dynamic cellular responses with PML phosphorylation, next, we investigated the significance of pPML S^518^ to the NBL disease trajectories and therapeutic vulnerability. High‐risk tumours (stages III and IV) demonstrated significant phosphorylation of PML (vs. stages I and II, *p* < .0001), highlighting its tumour‐suppressive function and cellular reprogramming capabilities those limit NBL progression (Figure 3B). Further, we observed that pPML S^518^ was significantly higher in metastatic (*p* = .0015 vs. primary; Figure 3C), relapsed (*p* = .0011) disease, suggesting its crucial role in driving cellular identity crisis that prompts metastasis and treatment‐refractory disease (Figure 3D). With the pPML S^518^ dynamics fingerprinted, we then investigated whether this phosphorylation is a key compensatory stress‐induced response to therapy pressure, augmenting cellular programs that mediate treatment evasion. Compared to disease at Dx, we observed a significant (*p* = .0005) phosphorylation of PML at S^518^ in PD (Figure 3E). Consistent with this, our analysis revealed a marked phosphorylation of PML at S^518^ in patients who received chemotherapy (*p* < .0001) and radiotherapy (*p* = .0083) reflecting (i) the selection or induction of adaptive resistance mechanisms under therapeutic pressure, and/or (ii) a failure of PML‐dependent tumour‐suppressive responses to therapy‐induced DNA damage resulting from PML destabilisation and functional disengagement (Figure 3F,G). Taken together, our findings identify a novel PTM that functions as a dynamic switch that promotes NBL progression and contributes to evolution of treatment resistant phenotypes.

### 3.4 PML S^518^ phosphorylation is inversely associated with PML availability

**FIGURE 4:**
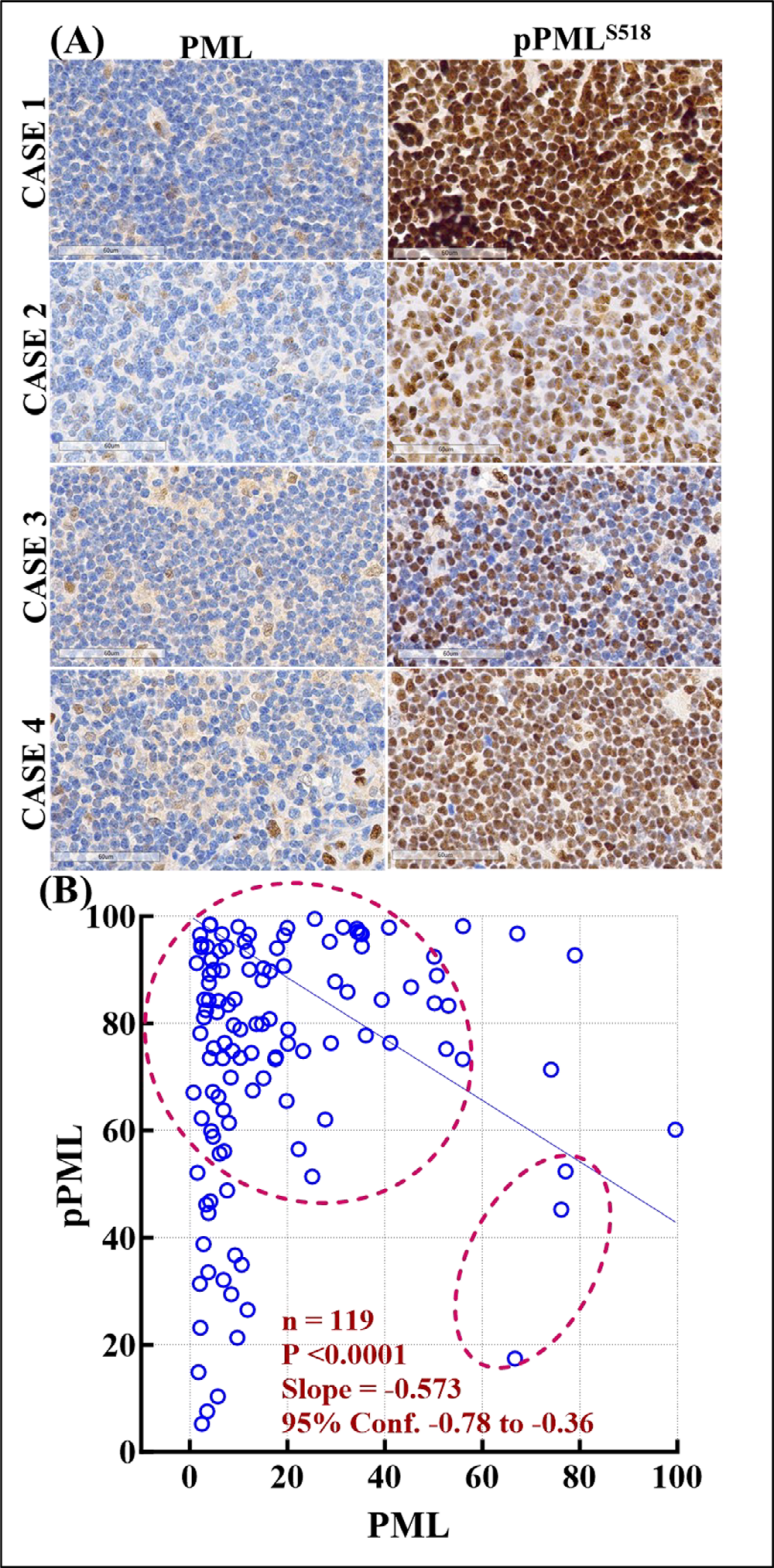
Promyelocytic leukaemia protein (PML) expression inversely correlates with phosphorylated promyelocytic leukaemia protein (pPML) S^518^ in neuroblastoma (NBL): (A) Representative microphotographs of matched NBL patients showing nuclear expression of PML and pPML S^518^ (Magnification 20×); (B) *XY* graph showing linear correlation (slope = −.573, *p* < .0001) between PML and pPML S^518^ expression in a cohort of 119 patients. PML and pPML S^518^ exhibit linear and inverse correlation in NBL patients.

With our realisation that PML S^518^ phosphorylation is a crucial post‐translational switch that governs the disease trajectory, we then investigated whether this phosphorylation corresponds to the PML loss. To reinforce S^518^ phosphorylation‐associated PML destabilisation paradigm in NBL patient cohort, we compared the expression profile of PML and its S^518^ phosphorylation status. In general, a remarkable PML loss with an increase in its phosphorylation at S^518^ and, high PML abundance in tumours where minimal S^518^ phosphorylation was evident. While the Pearson correlation is not significant (*p* = .0618), simple linear regression model displayed a significant (*p* < .0001) negative relationship (slope = −.573) between PML abundance and its site‐specific (S^518^) phosphorylation (Figure 4B). This inverse correlation between PML and pPML S^518^ indicates a definitive phosphorylation‐dependent PML destabilisation in NBL, and highlights a critical inflection point that diverge disease trajectory towards progression.

### 3.5 PML S^518^ phosphorylation predicts survival outcomes

**FIGURE 5:**
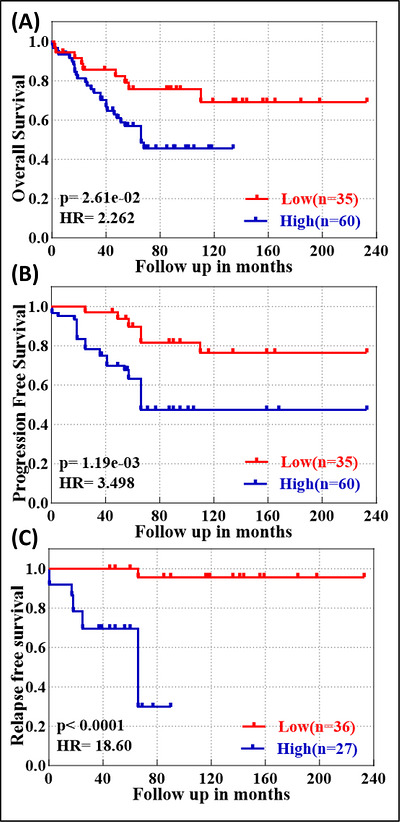
Association of high phosphorylated promyelocytic leukaemia protein (pPML) S^518^ with poor clinical outcomes: Kaplan–Meier curves showing the association of high pPML S^518^ with poor (A) overall survival (*n* = 95; hazard ratio [HR]: 2.262; 95% confidence interval [CI], 1.178–4.344); (B) progression‐free survival (*n* = 95; HR: 3.498; 95% CI, 1.774–6.900); and (C) relapse‐free survival in a cohort of 63 NBL patients (HR: 18.60; 95% CI, 6.941–49.84). All survival analysis (log‐rank Mantel‐Cox test and hazard ratio) and Kaplan‐–Meier curves were performed in GraphPad Prism. Blue = high pPML S^518^; Red = low pPML S^518^ expression.

Owing to the inverse association of S^518^ phosphorylation to the PML availability, we then examined its potential in serving as a predictive indicator for NBL clinical outcomes. Kaplan–Meier survival analysis integrated with log‐rank (Mantel–Cox) test in our cohort (*n* = 95) of NBL patients were constructed for survival (OS, RFS, PFS) with the high‐low (median threshold 74.50%) PML phosphorylation. To ensure comprehensive representation and to avoid potential bias, all tumour specimens available per patient, encompassing samples from multiple anatomical sites, as well as those collected from both Dx and PD were included. Of the 95 patients, 60 showed high while 35 patients showed low phosphorylation. Notably, elevated pPML S^518^ was consistently associated with adverse survival metrics including OS, PFS and RFS. Low pPML S^518^ was associated with better survival outcomes in contrast to high pPML S^518^ that showed adverse OS (*p* = 2.61e−02; HR: 2.262), predicting more than twofold risk of mortality (Figure 5A). More importantly, PFS in the high pPML S^518^ group was significantly low (*p* = 1.19e−03; HR: 3.498) with rapid disease progression, when compared with the low pPML S^518^ (Figure 5B). Furthermore, RFS was examined in a subset of 63 patients, where 27 were classified as high pPML S^518^ group and 36 as low pPML S^518^ group utilising the median (79.48%) S^518^ phosphorylation as the threshold. High pPML S^518^ levels decisively predict adverse RFS (*p* < .0001; HR: 18.60; Figure 5C). The distinct hazard ratio for RFS with high PML phosphorylation not only indicates a strong association but also predicts the increased risk of recurrence and mortality. Together, these outcomes establish S^518^‐specific PML phosphorylation as a clinically relevant prognostic marker associated with aggressive NBL behaviour and unfavourable patient outcomes. Given the persistence of elevated PML S^518^ phosphorylation in aggressive, therapy‐refractory tumours, we next explored the prognostic significance of this PTM within the same cohort of 53 treatment‐exposed patients. High levels of pPML‐S^518^ were strongly associated with unfavourable clinical outcomes across multiple survival measures. Patients with elevated pPML‐S^518^ exhibited significantly poorer OS (*p* = 1.83e−02; HR: 2.416; Figure S2D), indicating a greater risk of mortality compared with patients displaying lower phosphorylation levels. Increased pPML‐S^518^ was also associated with significantly reduced RFS (*p* = 4.07e−02; HR: 6.488; Figure S2E), highlighting a strong relationship between sustained PML phosphorylation and tumour recurrence following therapy. Although PFS did not reach statistical significance, a similar trend was observed, whereby elevated pPML‐S^518^ was associated with shorter PFS (*p* = 1.44e−01; HR: 2.098; Figure S2F). The magnitude and consistency of these associations suggest that persistent phosphorylation of PML at S^518^ may represent a critical molecular adaptation acquired during therapeutic stress, promoting the emergence of relapse‐competent and treatment‐resistant tumour states. Taken together, these findings establish pPML‐S^518^ as a clinically relevant biomarker of aggressive NBL behaviour, reflecting an adaptive signalling state that accompanies therapeutic resistance, disease recurrence, and poor patient prognosis across multiple survival endpoints. Critically, multivariable Cox proportional hazards analysis identified pPML S^518^ phosphorylation as an independent predictor of improved survival in patients with NBL (*p* = .039, Wald *χ* ^2^ = 4.256; Table 2C). Multicollinearity was assessed using VIF analysis, which demonstrated low VIF values for all covariates (Stage: 1.215; Age: 1.196; Gender: 1.054; MYCN status: 1.057), indicating minimal collinearity among predictors and supporting the robustness and stability of the Cox regression model (Table 2D). Collectively, these findings establish pPML S^518^ phosphorylation as an independent prognostic biomarker in NBL and suggest that its expression is associated with a more favourable clinical outcome. Furthermore, the observed loss of pPML S^518^ may reflect therapy‐adapted tumour evolution characterised by increased aggressiveness and disease progression, ultimately contributing to unfavourable clinical trajectories.

### 3.6 Prognostic significance of the pPML/PML ratio in patient survival outcomes

To determine whether the relative phosphorylation status of PML provides additional prognostic information beyond absolute PML or pPML expression levels, a pPML/PML ratio was calculated for each patient and used for survival analyses. Patients were stratified into low‐ and high‐ratio groups based on the mean ratio value. Kaplan–Meier analysis demonstrated that patients with a high pPML/PML ratio exhibited significantly poorer clinical outcomes compared with those with a low ratio (Figure S3). Specifically, a high pPML/PML ratio was associated with a reduction in OS, indicating that increased phosphorylation relative to total PML expression correlates with unfavourable patient prognosis (Figure S3A). Similarly, patients with a high pPML/PML ratio experienced significantly shorter RFS and PFS, suggesting an increased risk of disease recurrence and progression (Figure S3B,C). Notably, the prognostic associations observed with the pPML/PML ratio appeared more consistent across all three survival endpoints than analyses based on either total PML or pPML expression alone. These findings indicate that the relative phosphorylation status of PML, rather than absolute protein abundance, may better capture biologically relevant alterations in PML signalling associated with aggressive disease behaviour. Collectively, these results support the pPML/PML ratio as a potentially more informative prognostic biomarker, reflecting the balance between phosphorylated and total PML protein levels. The significant associations with OS, RFS, and PFS suggest that incorporation of this ratio into survival models may provide improved stratification of patient outcomes and further emphasise the importance of PML phosphorylation in disease progression.

### 3.7 S^518^ phosphorylation drives PML destabilisation and promotes an invasive phenotype in progressive NBL

**FIGURE 6:**
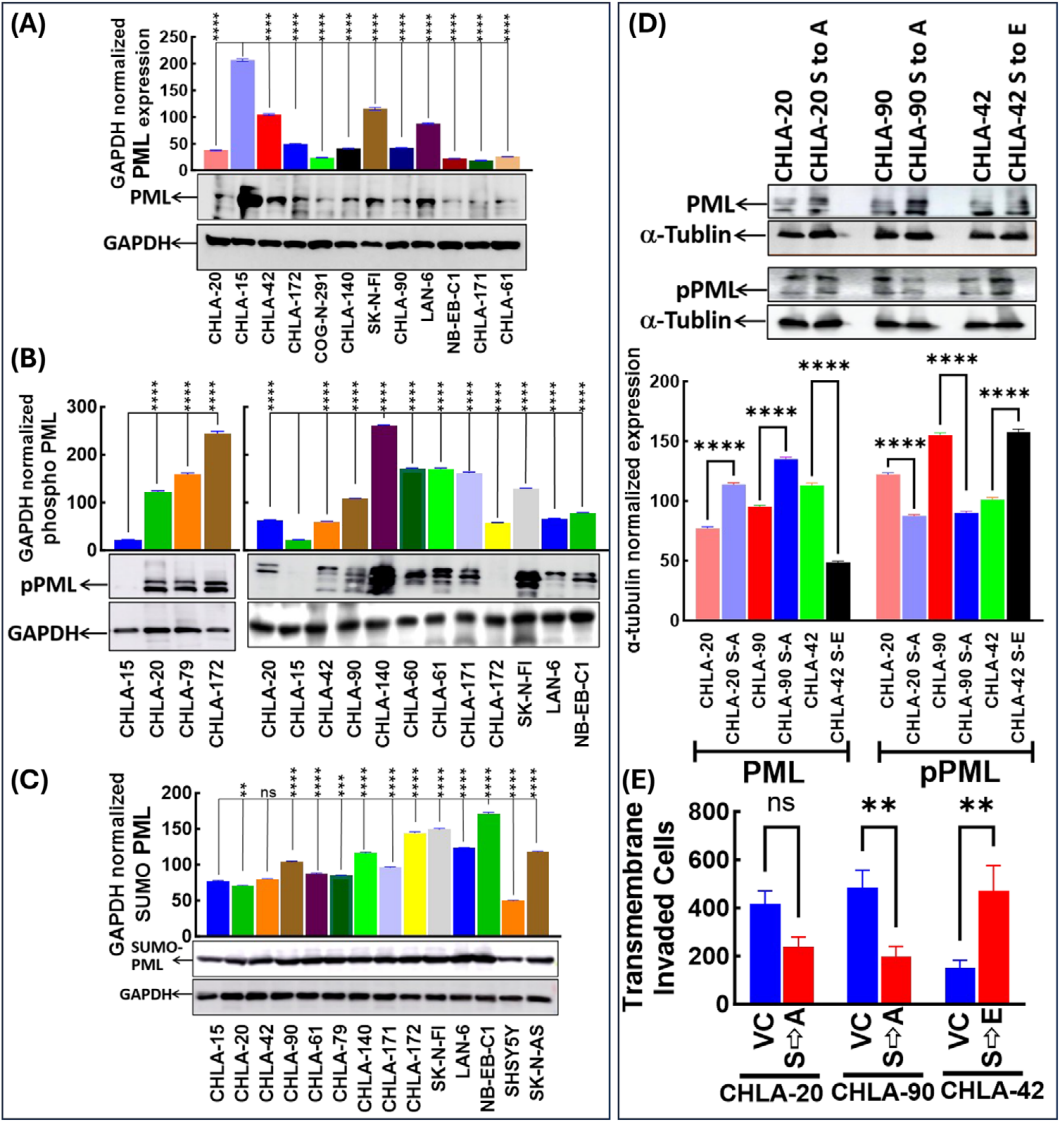
S^518^ phosphorylation promotes promyelocytic leukaemia protein (PML) destabilisation and is associated with enhanced invasive potential in high‐risk neuroblastoma (NBL). (A–C) Representative immunoblot and densitometric analyses of total PML (A), phospho‐PML S^518^ (B), and SUMOylation site‐specific PML (C) expression in a panel of stage 4 patient‐derived NBL cell lines established at diagnosis (Dx; CHLA‐15, CHLA‐42 and SH‐SY5Y) or at progressive disease (PD). Notably, CHLA‐15 and CHLA‐20 represent matched cell lines derived from the same patient at Dx and PD, respectively. PD‐derived NBL cells exhibited reduced total PML expression accompanied by increased S^518^ phosphorylation relative to Dx‐derived cells, consistent with enhanced PML turnover during disease progression. Expression of SUMOylation‐competent PML species was also altered in PD‐derived cells, suggesting coordinated regulation of PML post‐translational modification during NBL progression. (D) Immunoblot analysis of total PML and phospho‐PML expression following site‐directed mutagenesis of S^518^. Cells expressing the phospho‐deficient S518A mutant (serine‐to‐alanine) displayed enhanced PML protein stability and accumulation, whereas expression of the phosphomimetic S518E mutant (serine‐to‐glutamic acid) resulted in reduced PML abundance, demonstrating that phosphorylation at S^518^ promotes PML degradation. (E) Matrigel invasion assays performed in cells expressing wild‐type, S518A, or S518E PML constructs. Quantification of invading cells revealed that stabilisation of PML by the phospho‐deficient S518A mutation significantly suppressed tumour cell invasion, whereas the phosphomimetic S518E mutation enhanced invasive behaviour. These findings identify S^518^ phosphorylation as a critical post‐translational mechanism regulating PML stability and demonstrate that phosphorylation‐dependent PML degradation promotes the invasive phenotype of NBL cells.

To investigate the mechanistic role of PML S^518^ phosphorylation in NBL progression, we examined the expression of total PML, phosphorylated PML (S^518^), and SUMOylated PML in a panel of stage 4 NBL cell lines established from patients at Dx or PD. Analysis of these models revealed a distinct shift in PML post‐translational regulation during disease progression. Dx‐derived cell lines, including CHLA‐15 and CHLA‐42, displayed relatively high levels of total PML, whereas PD‐derived cell lines exhibited a marked reduction in PML protein abundance (Figure 6A). In contrast, phosphorylation of PML at S^518^ was consistently elevated in PD‐derived cells, demonstrating an inverse relationship between total PML expression and S^518^ phosphorylation (Figure 6B). This association was particularly evident in the matched CHLA‐15 and CHLA‐20 cell lines, which were established from the same patient at Dx and PD, respectively. Compared with CHLA‐15 cells, CHLA‐20 cells showed increased S^518^ phosphorylation accompanied by a substantial reduction in total PML, suggesting that acquisition of the progressive phenotype is associated with enhanced phosphorylation‐dependent PML turnover. Furthermore, analysis of SUMOylated PML species revealed concomitant alterations in PML PTM (Figure 6C), supporting the concept that coordinated phosphorylation and SUMOylation regulate PML stability during NBL progression.

Given the strong association between elevated S^518^ phosphorylation and reduced PML abundance, we next sought to determine whether phosphorylation at this residue directly regulates PML stability. To address this question, phospho‐deficient (S518A) and phosphomimetic (S518E) PML mutants were generated and expressed in NBL cells. Mutation of S^518^ to alanine, which prevents phosphorylation at this site, resulted in a pronounced accumulation of PML protein relative to wild‐type PML, indicating enhanced protein stability (Figure 6D). In contrast, substitution of S^518^ with glutamic acid, which mimics constitutive phosphorylation, caused a substantial reduction in PML protein levels (Figure 6D). These findings demonstrate that S^518^ phosphorylation is a critical determinant of PML protein turnover and establish a direct causal relationship between phosphorylation at this residue and PML degradation. Together with the expression patterns observed in PD‐derived NBL cells, these results support a model in which increased S^518^ phosphorylation drives loss of PML during tumour progression.

Because PML functions as a potent suppressor of tumour growth and dissemination, we next examined whether phosphorylation‐dependent regulation of PML stability influences the invasive behaviour of NBL cells. Matrigel invasion assays revealed that stabilisation of PML through expression of the phospho‐deficient S518A mutant significantly impaired tumour cell invasion compared with wild‐type controls (Figure 6E). Conversely, cells expressing the phosphomimetic S518E mutant exhibited enhanced invasive activity, indicating that constitutive phosphorylation‐dependent loss of PML promotes a more aggressive cellular phenotype (Figure 6E). The opposing effects of the S518A and S518E mutants on both PML abundance and invasive capacity strongly suggest that the biological consequences of S^518^ phosphorylation are mediated through regulation of PML stability. Importantly, these findings provide functional evidence that phosphorylation of PML at S^518^ is not merely a marker of disease progression but is a mechanistically important event required for PML destabilisation and the acquisition of invasive properties in NBL cells.

Collectively, these results identify S^518^ phosphorylation as a critical post‐translational switch that controls PML protein stability in NBL. Increased phosphorylation at this residue in PD promotes PML degradation, whereas prevention of phosphorylation stabilises PML and suppresses tumour cell invasion. These findings support a model in which aberrant activation of the S^518^ phosphorylation pathway contributes to NBL progression by functionally disabling the tumour‐suppressive activities of PML, thereby facilitating the transition to a more invasive and aggressive disease state.

## 4 Discussion

The present study uncovers a compelling and clinically relevant dimension of PML biology in NBL, a paediatric malignancy notorious for its aggressive behaviour and therapeutic resistance. While PML loss can be attributed to both transcriptional and post‐translational regulation, the absence of consistent evidence for widespread transcriptional silencing^12^, ^17^ has shifted the focus on proteasome‐mediated degradation as a more plausible explanation for PML loss. Although the ubiquitination and the consequence of proteasome‐degradation have been frequently reported the post‐translational priming mechanisms that are initiators and critical checkpoints of PML destabilisation still remain elusive.^5^, ^29^ By focusing on a unique PTM, phosphorylation at S^518^, this work delineates a mechanistic and prognostic framework that positions PML and its site‐specific phosphorylation as pivotal determinants of disease trajectory and clinical outcomes.

### 4.1 PML loss as a hallmark of aggressive NBL

Our findings demonstrate a striking downregulation of PML in high‐risk NBL tumours, particularly those with advanced stage, metastatic spread and relapse. These observations are consistent with a potential role for PML loss in disease progression and the disruption of cellular programs that maintain lineage fidelity and genomic stability. Notably, reduced PML expression was observed in therapy‐resistant tumours and post‐treatment specimens. While this finding may be consistent with the possibility that therapeutic pressure promotes adaptive cellular responses associated with PML destabilisation, our data may have limitations in establishing the definitive causal relationship. An alternative and potentially more direct interpretation is that PML is not effectively engaged as a downstream tumour suppressor in response to therapy‐induced DNA damage. In this context, persistent phosphorylation of PML at Ser^518^ may impair PML‐NB function, functionally uncoupling PML from its tumour‐suppressive activities and limiting appropriate cellular responses to chemotherapy‐ or radiotherapy‐induced genotoxic stress. These possibilities are not mutually exclusive and warrant further mechanistic investigation. Our findings are consistent with prior studies in solid tumours, including breast and lung cancers, where reduced PML expression has been associated with tumour aggressiveness and unfavourable clinical outcomes.^3^, ^12^, ^17^, ^35^, ^37^ Particularly in NBL, PML depletion or loss is frequently observed in metastatic tumours and is strongly associated with tumour recurrence, even in the absence of MYCN amplification.^9^ Mechanistic studies have demonstrated that PML suppresses tumour angiogenesis by upregulating thrombospondin‐2 (TSP2), a potent endogenous inhibitor of angiogenesis. Importantly, survival analyses in our cohort underscore the prognostic significance of PML availability.^9^ Patients with higher PML expression exhibited significantly improved OS, PFS, and RFS, supporting a protective role for PML in NBL. Furthermore, multivariate Cox regression identified PML as an independent predictor of survival, highlighting its potential utility as a biomarker for risk stratification in patients with NBL.

### 4.2 Phosphorylation at S^518^: A molecular switch for PML destabilisation

The most novel and impactful aspect of this study lies in the identification of S^518^ phosphorylation as a critical PTM that governs PML stability and function. Unlike other phosphorylation sites, S^518^ appears to serve as a unique signal integrator that primes PML for KLHL20–Cullin3‐mediated ubiquitination and proteasomal degradation.^4^ The inverse correlation between PML abundance and S^518^ phosphorylation, supported by regression analysis, suggests a direct mechanistic link whereby phosphorylation at this site destabilises PML‐NBs, thereby dismantling their regulatory architecture. This phosphorylation‐dependent loss of PML is not merely structural but functionally consequential. Tumours with elevated pPML S^518^ levels exhibited enhanced metastatic potential, and therapy resistance. The diffused nuclear localisation of pPML S^518^, in contrast to the punctate distribution of intact PML, further supports the notion of disrupted nuclear body integrity.^11^ These findings resonate with prior work in haematologic malignancies and prostate cancer, where phosphorylation‐driven degradation of PML has been shown to potentiate oncogenic signalling. Although the inverse relationship between PML abundance and pPML‐S^518^ narrowly missed the conventional threshold for statistical significance by Pearson correlation analysis (*p* = .0618), linear regression demonstrated a significant negative association between these variables (slope = −.573, *p* < .0001). This apparent difference likely reflects the substantial biological heterogeneity inherent to our clinically diverse NBL cohort, where inter‐patient variability may weaken the overall correlation coefficient despite the presence of a significant directional relationship. Importantly, we do not interpret these association analyses alone as evidence of causality. Rather, the clinical data support an inverse relationship between PML expression and S^518^ phosphorylation in NBL. To establish mechanistic relevance, we complemented these observations with functional phospho‐mutant studies. Across multiple NBL models, phospho‐deficient S518A mutants stabilised PML expression and suppressed aggressive cellular behaviour, whereas phosphomimetic S518E mutants reduced PML abundance and enhanced invasive phenotypes. Together, these findings suggest that S^518^ phosphorylation is not merely associated with reduced PML expression but functionally contributes to PML destabilisation and acquisition of aggressive disease characteristics, thereby supporting a mechanistic role for phosphorylation‐dependent regulation of PML during NBL progression.

### 4.3 Clinical implications and predictive value of PML and/or pPML S^518^

The clinical relevance of pPML S^518^ is underscored by its strong association with adverse survival outcomes. Patients with high levels of S^518^ phosphorylation faced significantly higher risks of mortality, rapid disease progression, and early relapse. The HR for RFS in particular were striking, suggesting that pPML S^518^ may serve as a robust predictive biomarker for recurrence. These findings are especially pertinent given the current therapeutic landscape of NBL, where relapse remains a major challenge despite aggressive multi‐modal treatment. Moreover, the study highlights a critical dependency of RA‐based differentiation therapy on PML integrity.^22^ Since RA signalling is mediated through PML‐NBs, phosphorylation‐induced PML loss may compromise the efficacy of this cornerstone therapy. This insight opens avenues for therapeutic intervention aimed at preserving PML stability or inhibiting its phosphorylation, thereby enhancing treatment responsiveness. To further evaluate whether total PML expression and pPML S^518^ provide independent prognostic information, we performed a combined Cox regression analysis including both markers in the same model. Because pPML S^518^ represents a phosphorylated form of PML, the two variables are biologically related and exhibited substantial overlap in their prognostic effects. In the combined analyses, PML remained significant while pPML S518 lost significance in one model, whereas the reverse was observed in an alternative model. These findings suggest that the prognostic information conveyed by PML and pPML S^518^ is largely overlapping rather than independent. Given the biological role of PML as the parent protein and the consistent association between loss of PML expression and adverse clinical outcomes, our results support the interpretation that PML loss is the more clinically relevant prognostic marker in this cohort. Nevertheless, the close relationship between PML and pPML S^518^ limits the ability to fully disentangle their individual contributions to prognosis, and this should be considered when interpreting these findings.

### 4.4 Broader biological significance and future directions

Beyond its immediate clinical implications, this study contributes to a broader understanding of how PTMs orchestrate tumour biology. The identification of a single site‐specific phosphorylation event as a master regulator of PML function exemplifies the precision with which cellular fate decisions are governed. It also raises intriguing questions about the upstream kinases responsible for S^518^ phosphorylation and the potential cross‐talk with other PTMs such as SUMOylation and ubiquitination. Future studies should aim to elucidate the signalling pathways that converge on S^518^, potentially involving stress‐responsive kinases or oncogenic drivers. Additionally, therapeutic strategies targeting the KLHL20–Cullin3 axis or modulating phosphorylation dynamics may offer novel avenues for restoring PML function in NBL. Unlike the proteasome inhibitors that indiscriminately block protein degradation and often induce systemic toxicity, such as neurotoxicity, growth impairment and immune dysfunction^18^, ^28^ that are particularly undesirable for paediatric patients, selectively targeting PTMs and the upstream regulators does not disrupt the homeostasis. The development of small molecule inhibitors or peptide mimetics that block S^518^ phosphorylation could represent a promising direction for translational research.

While this study provides novel insights into the role of PML and its site‐specific phosphorylation at S^518^ in NBL, several limitations should be acknowledged: (i) The study is based on a retrospective analysis of archived tumour specimens. Although the cohort was well annotated and diverse, retrospective designs inherently carry risks of selection bias and limited control over confounding variables. (ii) The majority of specimens were obtained from a single institution, which may limit the generalisability of the findings across broader populations. Multicentre validation cohorts would strengthen the external validity of the results. (iii) While the study establishes a strong correlation between PML S^518^ phosphorylation and clinical outcomes, direct functional assays to confirm causality, such as in vivo models manipulating S^518^ phosphorylation were not included. Our efforts are currently focused on these mechanistic studies to validate the role of S^518^ phosphorylation in driving therapy resistance and tumour progression. (iv) Although our study includes paired longitudinal samples collected from the same patients before and after therapy, unfortunately such samples are limited, precluding a robust paired‐sample analysis. Consequently, the present study relied on cross‐sectional comparisons between clinically annotated patient groups. Consequently, the observed differences in PML expression cannot be definitively attributed to treatment‐induced downregulation, as they may also reflect underlying heterogeneity in disease stage, tumour burden, risk status, or other clinical characteristics. Therefore, future studies utilising longitudinally collected paired samples will be necessary to determine whether therapeutic pressure directly contributes to PML loss and to clarify the temporal dynamics of PML regulation during NBL progression and treatment response. (v) Although the focus was on S^518^ phosphorylation, PML is subject to multiple PTMs (e.g., SUMOylation, acetylation, ubiquitination). The interplay between these modifications and their collective impact on PML function was not explored in this study. (vi) The classification of ‘high’ and ‘low’ expression/phosphorylation was based on median thresholds. While statistically sound, these cutoffs may not reflect biologically optimal thresholds for clinical decision‐making. Prospective studies with receiver operating characteristic (ROC) curve analysis could refine these metrics. (vii) Although the cohort was comprehensively annotated, certain clinicopathological variables, particularly treatment‐related parameters and disease stage, contained incomplete data, resulting in reduced sample sizes for specific multivariable models. To mitigate this issue, the final models were refined by restricting the number of covariates relative to the number of observed events and excluding variables that disproportionately contributed to model instability. While these steps improved model robustness, the potential for residual bias cannot be completely excluded. Therefore, validation in larger, independently annotated cohorts with more complete clinical data will be important to further refine risk estimates and confirm the generalisability of our findings. (viii) NBL is a paediatric cancer with unique developmental biology. The study does not fully explore how age‐related molecular contexts or developmental stage‐specific signalling pathways may influence PML regulation. Despite these limitations, the study provides a robust foundation for future investigations and highlights a promising biomarker axis with potential translational relevance in paediatric oncology.

Furthermore, our findings support a predominantly tumour‐suppressive association of PML in NBL, as reduced PML expression and increased phosphorylation at S^518^ were consistently linked to advanced disease, metastatic dissemination, relapse, therapy resistance, and unfavourable survival outcomes. Nevertheless, these observations should be interpreted within the broader framework of the increasingly recognised context‐dependent biology of PML. Although loss of PML correlates with poor prognosis in NBL, studies in other malignancies have demonstrated oncogenic functions for PML, including the promotion of cancer stem cell maintenance, metabolic fitness, cellular plasticity, and resistance to therapy.^1^, ^3^, ^10^, ^14^, ^15^, ^20^, ^21^, ^27^, ^38^ Therefore, our data do not imply a universally tumour‐suppressive role for PML across all cancers. Instead, they suggest that, within the NBL setting, preservation of PML abundance and nuclear body integrity is associated with a less aggressive clinical phenotype, whereas phosphorylation‐driven destabilisation of PML may facilitate tumour progression. These findings underscore the necessity of evaluating PML function in a disease‐specific context and highlight the importance of PTMs as critical determinants of its biological activity.

## 5 CONCLUSION

This study provides compelling evidence that the structural integrity and functional availability of PML are critical determinants of NBL progression, therapeutic response, and clinical outcomes. By focusing on a unique PTM, phosphorylation at S^518^, we reveal a mechanistic axis that governs PML destabilisation and nuclear body disassembly, thereby promoting therapy resistance and tumour evolution. Our findings demonstrate that PML loss is not merely a consequence of tumour evolution but a driver of aggressive disease phenotypes. The inverse relationship between PML expression and S^518^ phosphorylation underscores a phosphorylation‐dependent degradation pathway that dismantles the tumour‐suppressive functions of PML. This disruption compromises lineage fidelity, enhances metastatic potential, and facilitates the emergence of treatment‐refractory clones. Importantly, we establish that both PML loss and elevated pPML S^518^ levels are independently associated with poor survival outcomes, including reduced OS, rapid disease progression, and early relapse. These associations are statistically robust and clinically meaningful, positioning PML and its phosphorylation status as powerful prognostic and predictive biomarkers in NBL. Beyond prognostication, our study opens new therapeutic avenues. The dependency of RA‐based differentiation therapy on intact PML NBs suggests that stabilising PML or inhibiting its phosphorylation could enhance treatment efficacy. Targeting the KLHL20–Cullin3 ubiquitin ligase pathway or upstream kinases responsible for S^518^ phosphorylation may offer novel strategies to restore PML function and suppress tumour adaptability. In a broader context, this work contributes to the growing recognition of PTMs as master regulators of protein function and cellular fate. The identification of a single phosphorylation site as a decisive molecular switch exemplifies the precision of intracellular signalling mechanisms and their impact on cancer biology. Taken together, our study not only elucidates a previously underexplored facet of PML regulation in solid tumours but also provides a foundation for biomarker‐driven risk stratification and targeted therapeutic development in NBL. Future investigations should aim to validate these findings in larger, prospective cohorts and explore the translational potential of modulating PML phosphorylation as a strategy to combat high‐risk and relapsed NBL.

## AUTHOR CONTRIBUTIONS

Natarajan Aravindan contributed to the conception and design of the experiments. Sheeja Aravindan contributed to TMA preparation, and all immunohistochemistry experiments. Sreenidhi Mohanvelu, Madhi Oli Ramamurthy, Afsana Parveen Jahir Hussain contributed to the data analysis and interpretation. Sreenidhi Mohanvelu, Sheeja Aravindan, Sivaroopan Aravindan performed all digital pathology screening and quantification. Sreenidhi Mohanvelu, Natarajan Aravindan drafted the manuscript and Sivaroopan Aravindan, Poorvi Subramanian, Afsana Parveen Jahir Hussain, Madhi Oli Ramamurthy helped in revising it critically. All the authors read and approved the final manuscript.

## CONFLICT OF INTEREST STATEMENT

The authors declare no conflicts of interest.

## ETHICS STATEMENT

All protocols were approved by the University of Oklahoma Health Science Center (OUHSC) and Oklahoma State University (OSU) Institutional Review Boards (IRB), with permission for the research use of de‐identified specimens. All experiments were performed in accordance with our institutions’ IRB guidelines and regulations for the protection of human subjects.

## Supporting information




**Supporting Information File**: ctm270763‐sup‐0001‐figureS1.pptx

**Supporting Information File**: ctm270763‐sup‐0002‐figureS2.pptx

**Supporting Information File**: ctm270763‐sup‐0003‐figureS3.pptx

## Data Availability

The authors have nothing to report.
